# MALDI-TOF Mass Spectrometry as a Rapid Screening Alternative for Non-tuberculous Mycobacterial Species Identification in the Veterinary Laboratory

**DOI:** 10.3389/fvets.2022.827702

**Published:** 2022-01-28

**Authors:** Víctor Lorente-Leal, Emmanouil Liandris, Javier Bezos, Marta Pérez-Sancho, Beatriz Romero, Lucía de Juan

**Affiliations:** ^1^VISAVET Health Surveillance Center, Universidad Complutense de Madrid, Madrid, Spain; ^2^Animal Health Department, Faculty of Veterinary Medicine, Universidad Complutense de Madrid, Madrid, Spain

**Keywords:** MALDI-TOF MS, non-tuberculous mycobacteria (NTM), mycobacteria, Sanger sequencing, identification, veterinary samples, screening

## Abstract

Non-tuberculous mycobacteria (NTM) are difficult to identify by biochemical and genetic methods due to their microbiological properties and complex taxonomy. The development of more efficient and rapid methods for species identification in the veterinary microbiological laboratory is, therefore, of great importance. Although MALDI-TOF Mass Spectrometry (MS) has become a promising tool for the identification of NTM species in human clinical practise, information regarding its performance on veterinary isolates is scarce. This study assesses the capacity of MALDI-TOF MS to identify NTM isolates (*n* = 75) obtained from different animal species. MALDI-TOF MS identified 76.0% (*n* = 57) and 4% (*n* = 3) of the isolates with high and low confidence, respectively, in agreement with the identification achieved by Sanger sequencing of housekeeping genes (16S rRNA, *hsp65*, and *rpoB*). Thirteen isolates (17.3%) were identified by Sanger sequencing to the complex level, indicating that these may belong to uncharacterised species. MALDI-TOF MS approximated low confidence identifications toward closely related mycobacterial groups, such as the *M. avium* or *M. terrae* complexes. Two isolates were misidentified due to a high similarity between species or due to the lack of spectra in the database. Our results suggest that MALDI-TOF MS can be used as an effective alternative for rapid screening of mycobacterial isolates in the veterinary laboratory and potentially for the detection of new NTM species. In turn, Sanger sequencing could be implemented as an additional method to improve identifications in species for which MALDI-TOF MS identification is limited or for further characterisation of NTM species.

## Introduction

Mycobacteria are a diverse group of acid-fast Gram-positive bacilli that include more than 200 species differentiated into five newly emended genera (1). This group of ubiquitous bacteria can be found in a wide range of environments, with many species being important animal and human primary and secondary pathogens. Although the members of the *Mycobacterium tuberculosis* complex (MTBC), causal agents of tuberculosis (TB) in animals and humans, are probably the most widely studied mycobacterial agents in veterinary microbiology, other non-tuberculous mycobacteria (NTM), such as *Mycobacterium avium* subsp. *paratuberculosis* (MAP) in ruminants or *Mycobacterium marinum* in fish are also very relevant animal pathogens (2, 3). Importantly, NTM have been isolated from a diverse range of animals and, not only do they interfere with diagnostic methods implemented in the eradication and control of TB, but may also pose a risk for immunocompromised patients (4–6).

Molecular genetic methods greatly improved the capacity of clinical microbiology laboratories to identify NTM species with the development of several methods, such as genetic probe assays, including the INNO-LiPA Mycobacteria or the GenoType Common Mycobacteria assays (7–9). Although these methods are still used in many laboratories, they are expensive, identify a limited number of mycobacterial species and misidentify less prevalent NTM species due to probe cross-reactivity (10). By these reasons, sequence-based identification methods, such as Sanger sequencing, have become an alternative for the identification of mycobacterial species due to their nucleotide-level resolution as well as flexibility (11, 12). Advanced procedures based on Whole Genome Sequencing (WGS) have also been effectively used to identify NTM isolates and could be very useful for the description of novel NTM species or epidemiological investigations (13, 14). However, due to the increased costs and technical and training requirements of WGS, its implementation in routine diagnosis of NTM is limited. As a result, many laboratories still use a combination of probes and Sanger sequencing as a reference method to identify NTM species (8, 15, 16).

Sanger sequencing is based on the analysis of conserved genetic regions, mainly the hypervariable region of the 16S rRNA gene (11, 12, 17), the hypervariable region of the *hsp65* gene (18, 19), the *rpoB* gene (12, 20) or the 16S-23S internal transcribed spacer (21). Nevertheless, the routine use of sequencing in the microbiological laboratory is not as straightforward as it could initially seem for several reasons (11). Firstly, the ever-increasing number of mycobacterial species, taxonomical variations and high genetic similarity between species makes genetic identification a daunting task. In addition, the vast amount of sequence data in public repositories, which may not be appropriately annotated, and the rarity of certain mycobacterial species requires special caution when assigning an identification.

The introduction of MALDI-TOF MS in bacterial species identification has revolutionised clinical microbiology in the last decade, allowing a cost-effective and rapid identification of many important human pathogens (22). In the veterinary field, the application of MALDI-TOF MS has also received great interest and has been used to identify different veterinary important microbial pathogens, such as *Brucella* or *Staphylococcus*, with very promising results (23–25).

During many years, mycobacterial species have proven to be a difficult agent to identify through MALDI-TOF MS, mainly due to their complex cell wall composition and their fastidious growth. Since its first implementation in mycobacterial identification, there have been major advances in the use of MALDI-TOF MS for the identification of NTM species (26). In the first place, improvements in extraction protocols have enhanced the availability and quality of proteins for MALDI-TOF MS analysis (8, 27, 28). Secondly, an increasing number of mycobacterial spectra, which are the foundation for MALDI-TOF MS identification, have become available over the years (26, 29). Nevertheless, several mycobacterial species are still difficult to identify through proteomic methods, including veterinary important species, such as *M. bovis* or members of the *M. avium* complex (MAC). In this aspect, several efforts have been made to tackle these limitations, such as expanding Main Spectra Profiles (MSPs) of these mycobacteria for MALDI-TOF MS analyses (30, 31).

There is limited data regarding the general capacity of MALDI-TOF MS in the identification of NTM isolates in the veterinary setting, when compared to the information available in the human clinical practice (30–34). The aim of this study was to evaluate the use MALDI-TOF MS in the identification of NTM obtained from different animal species, and compare its performance against standard genetic methods.

## Materials and Methods

### Sample Selection and Processing

A total of seventy-five (*n* = 75) isolates were included in this study; 69 isolates were selected from a collection of samples obtained from animals (*n* = 20 species, Table 1) with suspected mycobacteriosis that were originally submitted for bacteriological culture during routine diagnostics by the Mycobacterial Unit of VISAVET Health Surveillance Centre (years 2011–2015). Six isolates corresponded to established reference strains and were used as controls (Table 1, Supplementary Table 1).

**Table 1:** Samples (*n* = 75) analysed in this study.

**N**°**samples**	**Animal species**	**Sample origin**
2	Alpaca	Lung, lymphnodes
1	American oystercatcher	Liver
32	Bovine	Lung, lymphnodes
1	Common shelduck	Lymphnodes
6	Deer	Lymphnodes
1	Domestic goat	Lymphnodes
2	Domestic Pig	Tissue homogenate, lymph nodes
1	Eurasian griffon	Lymphnodes
2	Ferret	Lymphnodes, liver, spleen
1	Fox	Tissue homogenate
1	Fulvous whistling duck	Liver, spleen
1	Giant Wood-Rail	Liver
1	Lesser kestrel	Lymphnodes
1	Mackerel	Necrotic granuloma
2	Malayan tapir	Trunk lavage
5	Mountain goat	Lymphnodes
1	Orangutan	Gastric lavage
1	Raccoon	Lymphnodes
6	Reference culture	Spanish type culture collection (CECT)
1	Roe Deer	Lymphnodes
7	Wild boar	Lymphnodes

*The relationship between the animal species and the isolates identified within these species can be found in Supplementary Table 1*.

All samples were originally considered as suspicious for tuberculosis and were therefore processed and cultured in the BSL3 facilities of VISAVET according to standardised mycobacteriological procedures as described elsewhere (35). Briefly, tissues were homogenised in sterile distilled water (Sigma-Aldrich, St. Louis, MO, USA), decontaminated with 0.75% (w/v) hexadecyl pyridinium chloride (Sigma-Aldrich) and centrifuged. Pellets were then swabbed on Löwenstein-Jensen and Coletsos slants supplemented with sodium pyruvate (Difco, Madrid, Spain), and incubated for a maximum of 3 months. When growth was observed, isolated colonies were analysed using PCR and DVR-spoligotyping in order to detect the presence of the MTBC (36, 37). Once the presence of MTBC members was discarded, a loop-full of mycobacterial culture was inoculated in 15 mL of Middlebrook 7H9 liquid medium, supplemented with sodium pyruvate and oleic albumin dextrose catalase (OADC) (Becton Dickinson, Franklin Lakes, NJ, USA), and incubated for up to 30 days. Cultures were visually inspected every 5, 10, 21, and 30 days and when microbial growth was visible, 1 and 1.5 mL of liquid culture were processed for proteomic and genetic analysis, respectively.

### Protein Extraction and MALDI-TOF MS Analysis

One mL of Middlebrook 7H9 medium was centrifuged at 15,500 × g for 2 min, the supernatant was discarded and the pellet was resuspended in 300 μL of HPLC-grade water (Sigma-Aldrich, St. Louis, MO, USA). Culture samples were then heat-inactivated at 100°C for 30 min and, following the addition of 900 μL of 96% ethanol (Panreac, Castellar del Vallès, Barcelona, Spain), these were stored overnight at −20°C.

Samples were centrifuged at 15,500 × g for 2 min, the supernatant was discarded and the pellet was let to air-dry at room temperature for at least 15 min. A small quantity of 0.5 mm glass beads and a volume equal to pellet size of HPLC-grade acetonitrile (Honeywell Fluka™, Charlotte, NC, USA) were added and samples were vortexed for 10 min and sonicated at 40 KHz for another 10 min on a Ultrasons sonicator (Selecta, Barcelona, Spain). An equal volume of 70% HPLC-grade formic acid (Sigma-Aldrich) was added and samples were centrifuged at 15,500 × g for 2 min. One μL of the supernatant was spotted on a polished steel target plate, let dry at room temperature and overlaid with one μL of α-cyano-4-hydroxy-cinnamic acid (HCCA) matrix HPLC grade (Bruker Daltonics, Billerica, MA, USA). Plates were then analysed on a Bruker Daltonik UltrafleXtreme MALDI TOF/TOF system (Bruker Daltonics) and spectra were compared against the Mycobacteria Library version 3.0 (38). Calibration of the Bruker BioTyper was performed using the Bruker Bacterial Test (BTS) according to the manufacturer's recommendations, and validation was achieved by running a reference strain of *E. coli* (ATCC 25922) as well as with the incorporation of mycobacterial reference strains (Table 1, Supplementary Table 1).

Final MALDI-TOF MS identifications were based on the score and consistency of the results. For the Bruker systems, MALDI-TOF scores higher or equal to 2.0 are required for a high confidence identification. Nevertheless, due to the biological features of mycobacteria, previous studies have shown that lower scores can effectively be used for NTM identification (15, 26). Therefore, MALDI-TOF scores higher or equal to 1.8 were considered high confidence identifications as described elsewhere (26) and were used to establish a positive NTM identification. Low confidence identifications were established when MALDI-TOF scores were obtained between 1.6 and 1.8, and non-reliable identifications were set when MALDI scores were below 1.6. In addition, consistency of the identification was evaluated by assessing the taxon and the ID score of the first five results returned by the MALDI-TOF MS. When more than one taxon was identified with a similar score, the results were approximated to the most related mycobacterial group. In the cases in which identifications were not reliable or had a low confidence score, extractions and MALDI-TOF MS analyses were repeated. In either case, these low confidence identifications were not considered for the final identification result.

### DNA Extraction and Sanger Sequencing

One and a half (1.5) mL of Middlebrook 7H9 media were centrifuged at 11,500 × g for 10 min. The pellet was washed in 1 mL of HPLC-grade water and samples were centrifuged again at 11,500 × g for 10 min. The pellet was then resuspended in 100 μL of HPLC-grade water and heat-inactivated at 100°C for 30 min.

**Figure 1:**
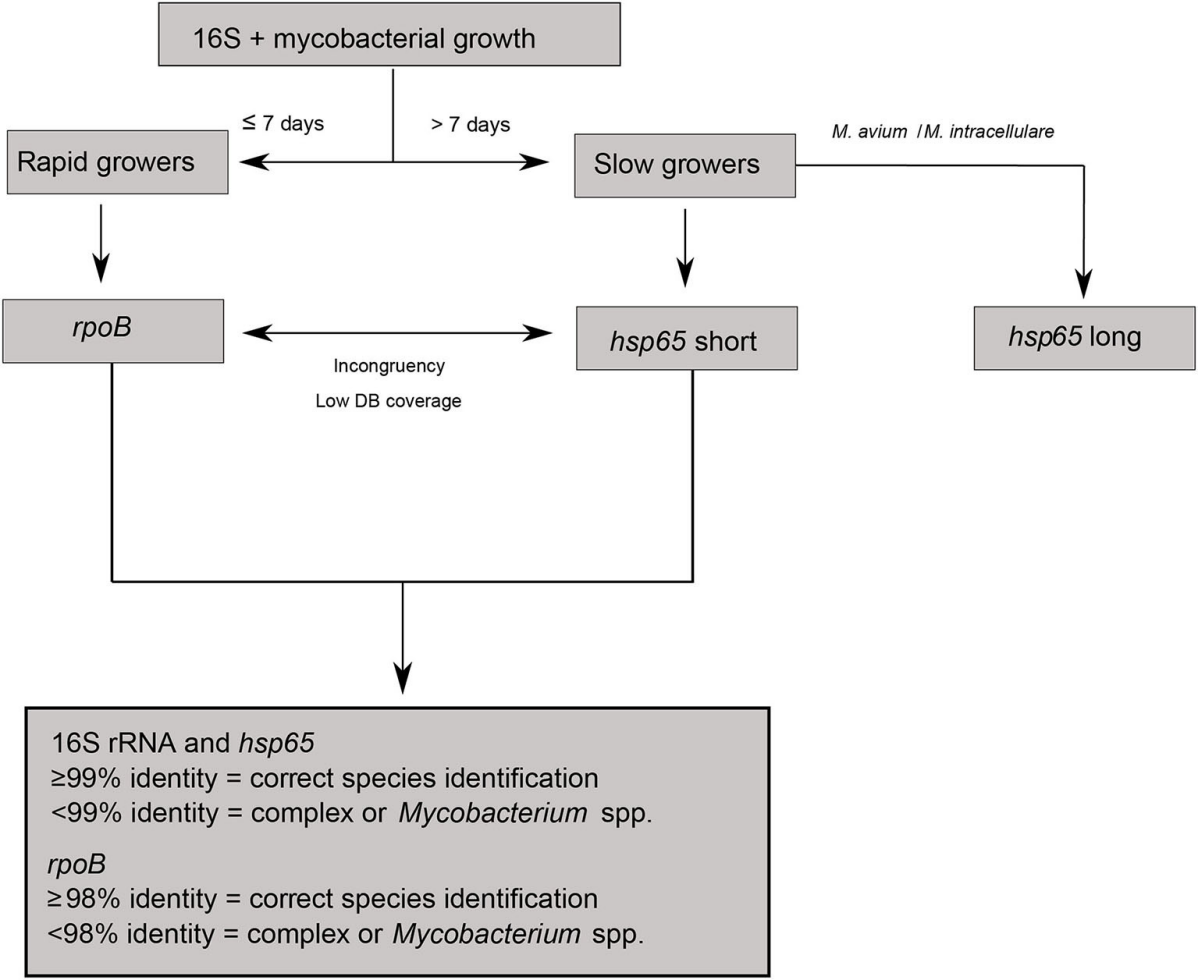
Algorithm describing species identification through Sanger sequencing.

For species identification through Sanger sequencing, samples were analysed following an algorithm designed according to growth speed in culture and partial 16S rRNA sequence (Figure 1) (17). The *rpoB* gene and the *hsp65* short fragment were used to identify rapid-growing and slow-growing mycobacteria, respectively (19, 20). In the case of *M. avium* and *M. intracellulare*, the *hsp65* long fragment was also used for species and subspecies identification (18). DNA amplicons were sent to STABvida (Lisbon, Portugal) for Sanger sequencing.

All DNA sequences were manually curated and compared against the NCBI nucleotide database using the Basic Local Alignment Search Tool (BLAST). Species identification was accepted when sequence identity was above 99% for the 16S rRNA and *hsp65* genes, and above 98% for the *rpoB* gene (11). If incongruent results were obtained between sequences or against MALDI-TOF MS identification, the *hsp65* short fragment or the *rpoB* gene were sequenced in those cases where they were not required initially (Figure 1). When sequence identity was low, identification was limited to the genus or complex level.

## Results

From the total number of isolates (*n* = 75), MALDI-TOF MS was able to identify 58 isolates (77.3%) with high confidence (score ≥ 1.8) to the group/species level, and 8 with low confidence (score between 1.6 and 1.8) (Table 2). The remaining isolates (*n* = 9) could not be identified reliably (score <1.6). One third (*n* = 25) of the total number of isolates were identified as *M. avium*.

**Table 2:** Species identification comparison between Sanger sequencing and MALDI-TOF MS (v3.0).

			**Score**			
**Isolate**	**Sanger sequencing ID**	**MALDI-TOF MS ID**	**<1.6**	**>1.6 and <1.8**	**≥1.8 and <2.0**	**>2.0**
17	*M. avium* subsp. *avium*	*M. avium*		2	4	11
8	‘*M. avium* subsp. *hominissuis*’	*M. avium*			6^*^	2
8	*M. avium* complex	*M. intracellulare/chimaera* group	8			
1	*Mycobacterium* spp^a^	*M. intermedium*	1			
2	*M. chitae*	*M. chitae*			1	1
1	*M. colombiense*	*M. colombiense*			1^*^	
1	*M. elephantis*	*M. elephantis*			1	
1	*M. engbaekii*	*M*.*hiberniae*/*engbaekii*^b^		1		
2	*M. europaeum*	*M. europaeum*			1	1
6	*M. fortuitum*	*M. fortuitum*			1	5
1	*M. fortuitum* complex	*M. fortuitum*		1		
1	*M. intracellulare*	*M. intracellulare/chimaera group*				1
1	*M. kansasii*	*M. kansasii*				1^*^
1	*M. malmesburyense*	*M. novocastrense*			1	
1	*M. neoaurum*	*M. neoaurum*		1		
4	*M. nonchromogenicum*	*M. nonchromogenicum*			1	3
1	*M. palustre*	*M. palustre*				1
3	*M. peregrinum*	*M. peregrinum*			1	2
2	*M. phlei*	*M. phlei*				2^*^
1	*M. septicum*	*M. septicum*			1	
1	*M. seoulense*	*M. seoulense*			1	
1	*M. shimoidei*	*M. shimoidei*			1	
1	*M. simiae* complex	*M*.*interjectum*^c^		1		
1	*M. smegmatis*	*M. smegmatis*				1
1	*M. terrae*	*M. terrae*				1^*^
2	*M. terrae complex*	*M*.*hiberniae*/*engbaekii*^b^		2		
4	*M. thermoresistibile*	*M. thermoresistibile*				4
1	*M. vaccae*	*M. vaccae*				1^*^

*Sanger sequencing identifications fulfilled the required thresholds depending on the genetic target used: 99% (16S rRNA and hsp65) and 98% (rpoB)*.

* *Includes a single reference strain (n = 6)*.

a *Closely related to M. bourgelatii and M. intermedium*.

b *M. terrae complex*.

c *M. simiae complex*.

The majority of the isolates (*n* = 62, 82.7%) were identified with high confidence to the species or subspecies level through Sanger sequencing (Table 2, Supplementary Table 1). One third (*n* = 25) of the total number of isolates were identified as *M. avium* subspecies, and could be further differentiated into *M. avium* subsp. *avium* (*n* = 17) and “*M. avium* subsp. *hominissuis*” (*n* = 8). The remaining non-*M. avium* isolates (*n* = 37) were identified as other NTM species, with most of them corresponding to *M. fortuitum* (*n* = 6)*, M. non-chromogenicum* (*n* = 4)*, M. thermoresistibile* (*n* = 4), and *M. peregrinum* (*n* = 3). Thirteen isolates could not be reliably identified to the species level based on Sanger sequencing, suggesting that they could possibly represent unknown NTM species. Sequence identity was below 98% for the complementary sequencing targets (i.e., *hsp65, rpoB* or both) for 12 isolates, but the 16S rRNA sequence was related to the *M. avium* (*n* = 8), *M. terrae* (*n* = 2), *M. fortuitum* (*n* = 1), or *M. simiae* (*n* = 1) complexes (Supplementary Table 1). An additional isolate could be classified as *M. bourgelatii* or *M. intermedium* based on 16S rRNA and *hsp65*/*rpoB* sequences, respectively. However, when compared to *M. intermedium*, this isolate presented the characteristic 12 bp gap of *M. bourgelatii* (data not shown).

The ability of the two methods to identify NTM was similar, since 88% (*n* = 66) of the isolates were identified by both methods (*n* = 57) or could not be identified by either (*n* = 9). In the latter case, non-reliable identifications obtained by MALDI-TOF MS corresponded to isolates for which Sanger sequencing could not provide a clear matching identification. In a similar manner to sequencing, MALDI-TOF MS approximated the identifications to closely related groups of species, such as the *M. avium* or *M. terrae* complexes (Supplementary Table 1).

The remaining isolates (*n* = 9) presented discording identifications and were evaluated further. One of these isolates was identified as *M. malmesburyense* by Sanger sequencing and as *M. novocastrense* through MALDI-TOF MS. Four isolates could be identified with Sanger sequencing but the MALDI-TOF MS identification had a low confidence score. Despite their low confidence score, the identification between three of these isolates agreed with the one obtained through Sanger sequencing, while the fourth isolate (*M. engbaekii*) could not be appropriately differentiated from *M. hiberniae*, a related species from the *M. terrae* complex. Finally, four isolates were identified with low confidence through MALDI-TOF MS but could only be identified to the complex level by Sanger sequencing.

## Discussion

The implementation of MALDI-TOF MS in the identification of mycobacteria has become an interesting alternative to genetic methods in human clinical microbiology laboratories. In veterinary mycobacteriology, MALDI-TOF has proven to be a reliable identification method of mycobacteria in a study evaluating 111 NTM isolates obtained from wild boars in Switzerland (33). In addition, other studies have focused on other veterinary important mycobacteria such as MAP in different animal hosts or *M. marinum* in fish (30, 34). Our study expands the utility of MALDI-TOF MS characterisation in a diverse array of animal species (*n* = 20), with an overall high agreement with Sanger sequencing.

Sanger sequencing is commonly used as a reference method for taxonomical identification of mycobacterial species in the clinical setting (8, 15, 16). However, its implementation is not without drawbacks due to the taxonomical complexity of the genus and the high genetic similarity of certain species or groups, which require the use of several housekeeping targets, and the lack of updated and curated sequence databases. Efforts have been made toward the automation of this process with the development of Open-Source tools for the identification of NTM species using Sanger sequencing data (12), which could greatly facilitate the use of this methodology in the future. In contrast, although WGS allow for a higher resolution than traditional sequencing of conserved genetic targets and show a great potential for the identification of NTM species directly from samples (14, 39), its increased costs and training requirements currently limits its implementation as a routine method in many microbiology laboratories. However, this may change in the future as WGS becomes more accessible with the development of simple bioinformatics tools.

MALDI-TOF MS offers a simplified process for NTM identification based on spectral similarities with a curated MSP database and reinforced with confidence values, leading to a more standardised procedure. In our study, high confidence MALDI-TOF MS scores (≥ 1.8) were used for NTM species identification, and the majority of the evaluated isolates were identified as such, indicating a good correlation with the stored MSPs in the database. A recent publication proposed the threshold for a high-confidence identification to be set at 2.0 (15). However, a threshold of 1.8 proved to be sufficient in the context of this study, since all identifications using this threshold agreed with the reference genetic method, with one exception (*M. malmesburyense*). Nevertheless, further studies including a wider range of NTM strains from different animal species should be carried out in the future to assess this threshold. Interestingly, identifications to the species or complex level could still be achieved even with low MALDI-TOF scores (1.6–1.8), indicating that these could provide valuable information that could be used by laboratory personnel to fine tune subsequent tests for isolate identification (15).

From the point of view of cost-effectiveness, MALDI-TOF MS presents several advantages to Sanger sequencing. Sanger sequencing requires at least two PCRs per isolate followed by, at least, two sequencing reactions per target. Therefore, 96 well-sequencing plates can analyse up to 48 samples per run, which in turn can take several hours. Furthermore, the obtained sequences require careful sequence curation and interpretation. In contrast MALDI-TOF MS requires just one procedure and up to 384 samples can be tested per run in a couple of minutes, allowing for a much more rapid identification and reduced cost per sample. Although our study used a liquid culture step from primary isolation until final identification, good identification results can also be achieved from primary isolations in solid media (26).

MALDI-TOF cannot effectively discern between certain closely related NTM species or subspecies of veterinary and human importance, such as *M. intracellulare* and *M. chimaera* or *M. avium* subspecies. These species represent an important group of mycobacteria in veterinary medicine that can be frequently isolated in cattle and swine (40–42). Their potential interference with routine animal TB diagnosis and their opportunistic nature make their identification an important element of eradication programmes and public health (2, 5, 43–45). Differentiation of these species can only be achieved through genetic methods, such as sequencing of the 3' end of the *hsp65* gene, PCR-based detection of specific Insertion Sequences or WGS approaches (18, 39, 46). One third of the isolates in our study corresponded to *M. avium* subspecies, and we additionally identified one *M. intracellulare-chimaera* group isolate. MALDI-TOF confidence scores in our study were, in general, high for these species, probably as a result of the large amount of MSPs stored in current libraries. This suggests that, despite its limit in resolution, MALDI-TOF MS could be used as a rapid method for initial screening of *M. avium* and therefore aid in the subsequent choice of the appropriate genetic targets for a more thorough characterisation. Recent efforts in the analysis of MSPs from this group of bacteria suggest that, with an appropriately curated database, MALDI-TOF MS identification of these, and other, challenging species could be achievable in the future (30, 31, 47). Certainly, MSP analysis for the detection of characteristic peaks between closely related mycobacteria should be carried out in the future (48). In addition, the analysis of lipid profiles through positive-ion MALDI-TOF MS or alternative MS instruments, such as High Resolution Tandem Mass Spectrometry, have been recently used to differentiate between closely related mycobacteria, such as the MTBC and *M. abscessus* subspecies (48, 49). Although these studies were carried out in a limited number of strains, their use in other closely related mycobacteria could also be an interesting alternative in the future.

Misidentifications and non-reliable identifications are two important limitations in the implementation of MALDI-TOF MS in microbiological laboratories (33). This is mainly a result of a reduced number and variety of MSPs for less prevalent mycobacterial species in current databases or the absence of MSPs for unknown NTM species.

Low confidence and non-reliable identifications in our study were mostly observed in isolates that could not be identified through Sanger sequencing either. Interestingly, although MALDI-TOF identifications in these cases are not considered reliable, they were similar to the ones obtained through Sanger sequencing. For example, the isolate that was closely related to *M. bourgelatii/intermedium* was identified as *M. intermedium* by MALDI-TOF MS, and the *M. simiae* complex isolate was identified as *M. interjectum*, a closely related species as seen by sequencing of 16S rRNA and *rpoB* genes. This indicates that MALDI-TOF may also be useful in detecting non-established NTM species and aid in the selection of complementary methods for a cost-effective characterisation (50). Further characterisation of these isolates would be needed to define these species and is outside of the scope of this publication.

Only one misidentification with a high-confidence score was observed in our study, which corresponded to *M. malmesburyense*, a rare mycobacterial species for which no MSPs are currently available. In addition to the absence of MSPs, a limited number of spectra from less prevalent mycobacterial species (e.g., *M. setense*) has also been shown to have a negative effect in MALDI-TOF MS performance (15, 29). The isolation of rare and unknown NTM species in animal samples in this study, as well as in the one published by Ghielmetti et al., strongly indicate that animals contain a large diversity of NTM species that is not being represented by current nucleotide and MSP databases, which are mainly focused on human clinical isolates (24, 33). Furthermore, as more animal and environmental sources are sampled, there is an increasing need to describe novel species of mycobacteria for a better classification and understanding of mycobacterial ecology and taxonomy. Thus, the addition of MSPs from animal sources could aid in the identification of less prevalent mycobacterial species and improve the performance of MALDI-TOF MS identification in the future.

In conclusion, despite the limited discriminatory power among certain mycobacterial groups, MALDI-TOF MS may be a suitable alternative for NTM species identification for several reasons. Unlike sequencing, in which multiple targets need to be identified, purified and reprocessed with a sequencing PCR, MALDI-TOF spectra are readily obtained after the extraction protocol. In addition, MALDI-TOF steel plates allow for the simultaneous analysis of a large number of samples, making this methodology extremely time- and cost-efficient. If adequate MSP libraries are available, data processing is minimal, and does not require thorough taxonomic or database research which could lead to erroneous species identification (11). However, there is a need to incorporate a larger number of MSPs from veterinary isolates in the available databases in order to increase MALDI-TOF performance with samples with a high diversity of mycobacterial species, such as those originating from animals or environmental sources. Nevertheless, even in the absence of curated MSPs, MALDI-TOF can be a powerful tool for the detection of potentially unknown NTM species. In these cases, or when closely-related mycobacterial species are detected, the use of more precise molecular genetic methods could be of use and refined identification algorithms could significantly improve identification turnaround times.

## Data Availability Statement

The datasets presented in this study can be found in online repositories. The names of the repository and accession numbers can be found at: https://www.ncbi.nlm.nih.gov/nuccore/, OK538881 - OK538918, OK539021 - OK539047, OK538946 - OK539020, OK538919 - OK538945.

## Ethics Statement

Ethical review and approval was not required for the animal study because all animals were sampled due to suspicion of mycobacterial infection in a clinical setting or under an official eradication programme (e.g., tuberculosis). Written informed consent for participation was not obtained from the owners because all animals were sampled due to suspicion of mycobacterial infection in a clinical setting or under an official eradication programme (e.g., tuberculosis).

## Author Contributions

VL-L performed all the experiments in this study. MP-S, EL, and BR participated in the design of the study. LJ, JB, and BR were responsible for obtaining the animal samples. VL-L wrote the manuscript with the insights of all the co-authors. BR and MP-S supervised the study. All authors contributed to the article and approved the submitted version.

## Funding

VL-L was funded with a predoctoral grant from the Complutense University of Madrid and Banco Santander 2017–2018. This work was partially financed and supported by the Ministerio de Agricultura, Pesca y Alimentación and the Programa de Tecnologías Avanzadas en Vigilancia Sanitaria (TAVS) de la Comunidad de Madrid (S2013/ABI2747). This work was partially funded by the European Union Reference Laboratory for bovine tuberculosis.

## Conflict of Interest

The authors declare that the research was conducted in the absence of any commercial or financial relationships that could be construed as a potential conflict of interest.

## Publisher's Note

All claims expressed in this article are solely those of the authors and do not necessarily represent those of their affiliated organizations, or those of the publisher, the editors and the reviewers. Any product that may be evaluated in this article, or claim that may be made by its manufacturer, is not guaranteed or endorsed by the publisher.
