# Supplementary material for: MALDI-TOF Mass Spectrometry as a Rapid Screening Alternative for Non-tuberculous Mycobacterial Species Identification in the Veterinary Laboratory
Source: Front Vet Sci. 2022 Jan 28;9:827702. doi: 10.3389/fvets.2022.827702 (PMC8831857; doi:10.3389/fvets.2022.827702)
Supplement: Supplementary file 1 [file Data_Sheet_1.PDF]

Supplementary Material

Supplementary Table 1. Species identification of the NTM species isolated in this study. In cases where sequence ID was not reliable (<99% or <98%), closest related mycobacterial complex or species is specified. ID = Identification. N.D. = Not Done.

Code	Origin	16S	Hsp65 short	Hsp65 long	RpoB	Final ID
19	Mountain goat	*M. avium* complex	Not reliable ID (<98%)[1]	N.D.	Not reliable ID (<98%)[2]	*M. avium* complex
20	Alpaca	*M. avium* complex	Not reliable ID (<98%)[1]	N.D.	Not reliable ID (<98%)[2]	*M. avium* complex
27	Alpaca	*M. avium* complex	Not reliable ID (<98%)[1]	N.D.	Not reliable ID (<98%)[2]	*M. avium* complex
45	Deer	*M. avium* complex	Not reliable ID (<98%)[1]	N.D.	Not reliable ID (<98%)[2]	*M. avium* complex
58	Roe Deer	*M. avium* complex	Not reliable ID (<98%)[1]	N.D.	Not reliable ID (<98%)[2]	*M. avium* complex
103	Bovine	*M. avium* complex	Not reliable ID (<98%)[1]	N.D.	Not reliable ID (<98%)[2]	*M. avium* complex
110	Wild boar	*M. avium* complex	Not reliable ID (<98%)[1]	N.D.	Not reliable ID (<98%)[2]	*M. avium* complex
147	Bovine	*M. avium* complex	Not reliable ID (<98%)[1]	N.D.	Not reliable ID (<98%)[2]	*M. avium* complex
9	Deer	*M. avium*	N.D.	*M. avium* subsp. *avium*	N.D.	*M. avium* subsp. *avium*
14	Fulvous whistling duck	*M. avium*	N.D.	*M. avium* subsp. *avium*	N.D.	*M. avium* subsp. *avium*
16	Common shelduck	*M. avium*	N.D.	*M. avium* subsp. *avium*	N.D.	*M. avium* subsp. *avium*
22	Mountain goat	*M. avium*	N.D.	*M. avium* subsp. *avium*	N.D.	*M. avium* subsp. *avium*
23	Mountain goat	*M. avium*	N.D.	*M. avium* subsp. *avium*	N.D.	*M. avium* subsp. *avium*
24	Mountain goat	*M. avium*	N.D.	*M. avium* subsp. *avium*	N.D.	*M. avium* subsp. *avium*
25	Eurasian griffon	*M. avium*	N.D.	*M. avium* subsp. *avium*	N.D.	*M. avium* subsp. *avium*
29	American oystercatcher	*M. avium*	N.D.	*M. avium* subsp. *avium*	N.D.	*M. avium* subsp. *avium*
51	Lesser kestrel	*M. avium*	N.D.	*M. avium* subsp. *avium*	N.D.	*M. avium* subsp. *avium*
52	Mountain goat	*M. avium*	N.D.	*M. avium* subsp. *avium*	N.D.	*M. avium* subsp. *avium*
53	Domestic goat	*M. avium*	N.D.	*M. avium* subsp. *avium*	N.D.	*M. avium* subsp. *avium*
54	Deer	*M. avium*	N.D.	*M. avium* subsp. *avium*	N.D.	*M. avium* subsp. *avium*
59	Deer	*M. avium*	N.D.	*M. avium* subsp. *avium*	N.D.	*M. avium* subsp. *avium*
62	Domestic pig	*M. avium*	N.D.	*M. avium* subsp. *avium*	N.D.	*M. avium* subsp. *avium*
98	Raccoon	*M. avium*	N.D.	*M. avium* subsp. *avium*	N.D.	*M. avium* subsp. *avium*
129	Bovine	*M. avium*	N.D.	*M. avium* subsp. *avium*	N.D.	*M. avium* subsp. *avium*
154	Bovine	*M. avium*	N.D.	*M. avium* subsp. *avium*	N.D.	*M. avium* subsp. *avium*
2	CECT 7407	*M. avium*	N.D.	*M. avium* subsp. *hominissuis*	N.D.	*M. avium* subsp. *hominissuis*
15	Ferret	*M. avium*	N.D.	*M. avium* subsp. *hominissuis*	N.D.	*M. avium* subsp. *hominissuis*
57	Ferret	*M. avium*	N.D.	*M. avium* subsp. *hominissuis*	N.D.	*M. avium* subsp. *hominissuis*
81	Bovine	*M. avium*	N.D.	*M. avium* subsp. *hominissuis*	N.D.	*M. avium* subsp. *hominissuis*
104	Domestic pig	*M. avium*	N.D.	*M. avium* subsp. *hominissuis* .	N.D.	*M. avium* subsp. *hominissuis*
114	Wild boar	*M. avium*	N.D.	*M. avium* subsp. *hominissuis*	N.D.	*M. avium* subsp. *hominissuis*
125	Fox	*M. avium*	N.D.	*M. avium* subsp. *hominissuis*	N.D.	*M. avium* subsp. *hominissuis*
144	Bovine	*M. avium*	N.D.	*M. avium* subsp. *hominissuis*	N.D.	*M. avium* subsp. *hominissuis*

[1] closely related to *M. scrofulaceum*

[2] closely related to *M. bouchedurhonense*

Supplementary Table 1 (continued).

Code	Origin	16S	Hsp65 short	Hsp65 long	RpoB	Final ID
61	Bovine	*M. bourgelatii*	*M. intermedium*	N.D.	*M. intermedium*	*Mycobacterium* sp.[3]
55	Wild boar	*M. chitae*	*M. chitae*	N.D.	N.D.	*M. chitae*
75	Bovine	*M. chitae*	*M. chitae*	N.D.	N.D.	*M. chitae*
6	CECT 3035	*M. avium* complex	*M. colombiense*	N.D.	N.D.	*M. colombiense*
117	Wild boar	*M. engbaekii*	N.D.	N.D.	*M. engbaekii*	*M. engbaekii*
44	Deer	M. europaeum	M. europaeum/ scrofulaceum	N.D.	*M. europaeum*[4]	*M. europaeum*
161	Bovine	*M. europaeum*	*M. europaeum*	N.D.	N.D.	*M. europaeum*
5	Malayan tapir	*M. fortuitum*	N.D.	N.D.	*M. fortuitum*	*M. fortuitum*
21	Malayan tapir	*M. fortuitum*	N.D.	N.D.	*M. fortuitum*	*M. fortuitum*
28	Bovine	*M. fortuitum*	N.D.	N.D.	*M. fortuitum*	*M. fortuitum*
31	Ipecaa	*M. fortuitum*	N.D.	N.D.	*M. fortuitum*	*M. fortuitum*
149	Bovine	*M. fortuitum*	N.D.	N.D.	*M. fortuitum*	*M. fortuitum*
156	Bovine	*M. fortuitum*	N.D.	N.D.	*M. fortuitum*	*M. fortuitum*
56	Bovine	*M. fortuitum* complex	N.D.	N.D.	*M. septicum*	*M. septicum*
157	Bovine	*M. intracellulare/chimerae*	N.D.	*M. intracellulare*	N.D.	*M. intracellulare*
1	CECT 3030	*M. kansasii /gastri*	*M. kansasii*	N.D.	N.D.	*M. kansasii*
80	Bovine	*M. malmesburyense*	N.D.	N.D.	*M. malmesburyense*	*M. malmesburyense*
109	Bovine	*M. neoaurum*	N.D.	N.D.	*M. neoaurum*	*M. neoaurum*
105	Bovine	*M. nonchromogenicum*	*M. nonchromogenicum*	N.D.	N.D.	*M. nonchromogenicum*
121	Bovine	*M. nonchromogenicum*	*M. nonchromogenicum*	N.D.	N.D.	*M. nonchromogenicum*
128	Bovine	*M. nonchromogenicum*	*M. nonchromogenicum*	N.D.	N.D.	*M. nonchromogenicum*
131	Wild boar	*M. nonchromogenicum*	*M. nonchromogenicum* (98%)	N.D.	*M. nonchromogenicum*	*M. nonchromogenicum*

[3] Closely related to *M. bourgelatii* and *M. intermedium*

[4] Isolate with 99% identity and triplet nucleotide substitution

Supplementary Table 1 (continued).

N	Origin	16S	Hsp65 short	Hsp65 long	RpoB	Final ID
73	Bovine	*M. palustre*	*M. palustre*	N.D.	N.D.	*M. palustre*
159	Bovine	*M. simiae* complex[5]	Not reliable ID (<99%)[6]	N.D.	Not reliable ID (<98%)[7]	*M. simiae* complex
113	Bovine	*M. fortuitum* complex	N.D.	N.D.	*M. peregrinum*	*M. peregrinum*
68	Bovine	*M. fortuitum* complex	N.D.	N.D.	*M. peregrinum*	*M. peregrinum*
143	Bovine	*M. fortuitum* complex	N.D.	N.D.	*M. peregrinum*	*M. peregrinum*
10	CECT 3009	*M. phlei*	N.D.	N.D.	*M. phlei*	*M. phlei*
146	Bovine	*M. phlei*	N.D.	N.D.	*M. phlei*	*M. phlei*
47	Bovine	*M. fortuitum* complex	Not reliable ID (<99%)[8]	N.D.	Not reliable ID (<98%)[9]	*M. fortuitum* complex
158	Bovine	*M. shimoidei*	*M. shimoidei*	N.D.	N.D.	*M. shimoidei*
107	Bovine	*M. smegmatis*	N.D.	N.D.	*M. smegmatis*	*M. smegmatis*
12	CECT 3028	*M. terrae*	*M. terrae*	N.D.	N.D.	*M. terrae*
115	Wild boar	*M. engbaekii*	*M. arupense*	N.D.	Not reliable ID (<98%)[10]	*M. terrae* complex
118	Wild boar	*M. engbaekii*	*M. arupense*	N.D.	Not reliable ID (<98%)[10]	*M. terrae* complex
13	Orangutan	*M. thermoresistibile*	N.D.	N.D.	*M. thermoresistibile*	*M. thermoresistibile*
71	Bovine	*M. thermoresistibile*	N.D.	N.D.	*M. thermoresistibile*	*M. thermoresistibile*
79	Bovine	*M. thermoresistibile*	N.D.	N.D.	*M. thermoresistibile*	*M. thermoresistibile*
82	Bovine	*M. thermoresistibile*	N.D.	N.D.	*M. thermoresistibile*	*M. thermoresistibile*
30	CECT 3019	*M. vaccae*	N.D.	N.D.	*M. vaccae*	*M. vaccae*
169	Mackerel	*M. elephantis*	N.D.	N.D.	*M. elephantis.*	*M. elephantis*
26	Deer	*M. seoulense/ paraseoulense*	*M. seoulense/ paraseoulense*	*N.D.*	*M. seoulense*	*M. seoulense*

[5] Closely related to *M. paraense, M. interjectum* and *M. saskatchewanense*.

[6] Related to *M. paraense*.

[7] Related to *M. paraense* and *M. interjectum.*

[8] Related to *M. peregrinum, M. mortmartrense* and *M. septicum*.

[9] Related to *M. fortuitum*.

[10] Related to *M. engbaekii*
